# Motor organization of unilateral polymicrogyria associated with ipsilateral brainstem atrophy – a case report

**DOI:** 10.1186/s12883-022-02795-y

**Published:** 2022-08-18

**Authors:** Choong-Hee Roh, Da-Sol Kim, Gi-Wook Kim, Yu Hui Won, Myoung-Hwan Ko, Jeoung-Hwan Seo, Sung-Hee Park

**Affiliations:** 1 Department of Physical Medicine & Rehabilitation, Jeonbuk National University Hospital, 20, Geonji-ro, Deokjin-gu, Jeonju-si, Jeollabuk-do 54907 Republic of Korea; 2 Research Institute of Clinical Medicine of Jeonbuk National University - Biomedical Research Institute of Jeonbuk National University Hospital, Jeonju, 54907 Republic of Korea

**Keywords:** Polymicrogyria, Transcranial magnetic stimulation, Diffusion tensor imaging, Mirror movement, Congenital hemiplegia

## Abstract

**Background:**

Polymicrogyria refers to the disruption of normal cerebral cortical development late in neuronal migration or in early cortical organization. Although patients with polymicrogyria feature relatively favorable motor outcomes, polymicrogyric lesions accompanied by extensive unilateral hemispheric atrophy and ipsilateral brainstem atrophy may induce poorer motor outcomes. This study is the first to employ transcranial magnetic stimulation (TMS) and diffusion tensor imaging (DTI) to characterize changes to motor organization and white matter tracts induced by polymicrogyria.

**Case presentation:**

We document a case of a 16-year-old female with left hemiplegic unilateral polymicrogyria associated with ipsilateral brainstem atrophy. Magnetic resonance imaging (MRI) of the brain revealed unilateral polymicrogyria to have affected anterior cortical areas, including the perisylvian region on the right side. The right halves of the brain and brainstem were significantly smaller than the left halves. Although our patient was found to exhibit cortical dysplasia of the right frontoparietal and sylvian fissure areas and a decreased number of fibers in the corticospinal tract (CST) of the affected side on DTI, the connectivity of the CST was preserved up to the motor cortex. We also measured the cross-sectional area of the CST at the level of the pons. In TMS, contralateral motor evoked potentials (MEPs) were evoked from both hands, but the ipsilateral MEPs were evoked only from the left hand. The left hand featured a long duration, polyphasic pattern of contralateral MEPs.

**Discussion and conclusion:**

TMS revealed that the concurrent bilateral projections to the paretic hand from the affected and unaffected hemispheres and contralateral MEPs in the paretic hand were polyphasic, indicating delayed electrophysiological maturation or a pathologic condition of the corticospinal motor pathways. In DTI, the cross-sectional area of the CST at the level of the pons on the affected side was smaller than that on the unaffected side. These DTI findings reveal an inadequate CST volume. Despite extensive brain malformation and ipsilateral brainstem atrophy, our patient had less severe motor dysfunction and presented with involuntary mirror movements. Mirror movements in the paretic hand are considered to indicate ipsilateral corticospinal projections from the unaffected hemisphere and may suggest favorable motor outcomes in early brain injury.

## Background

Polymicrogyria refers to the disruption of normal cerebral cortical development late in neuronal migration or in early cortical organization [1]. While the range and severity of its clinical manifestations depend on the extent of cortical involvement, previous research has associated a homogenous phenotype of seizure disorders, spastic hemiplegia, and intellectual disability with unilateral polymicrogyria and ipsilateral brainstem hemiatrophy. The consistency of this association suggests a new clinical entity: “hemi-microencephaly” or “unilateral microencephaly.” [2]. A detailed study of the motor organization corresponding to this type of brain lesion has not been performed, and whether this clinical entity induces hemiplegic weakness remains unclear.

Although patients with polymicrogyria display relatively favorable motor outcomes, polymicrogyric lesions accompanied by extensive unilateral hemispheric atrophy and ipsilateral brainstem atrophy may induce poorer motor outcomes. Herein, we document the case of a congenital left hemiplegic patient with unilateral cerebral polymicrogyria associated with ipsilateral brainstem atrophy. Despite extensive brain malformation and ipsilateral brainstem atrophy observed via brain imaging, the patient exhibited less severe motor dysfunction than expected and presented with involuntary mirror movements in the paretic hand. Previous studies have reported the usefulness of TMS for the evaluation of pathological conditions in the motor cortex of the brain [3]. Furthermore, DTI has been found to be useful for visualizing the corticospinal tract (CST) and assessing the integrity of neural fibers [4]. Therefore, in this study, we used TMS and DTI to characterize changes in motor organization and white matter tracts induced by polymicrogyria.

## Case presentation

Our patient presented with left hemiparesis from infancy with mirror movements in her paretic hand. She was born at full-term via normal delivery and without any perinatal complications. No relevant family history was reported. She had delayed developmental milestones. At the age of 2, she began walking, and she experienced complex partial motor seizures and secondary generalized tonic–clonic seizures which were controlled with 200 mg topiramate per day. Electroencephalography examination revealed frequent spikes in the right frontotemporal area. She could perform daily activities with her paretic hand but experienced decreased pinch strength and dexterity in the left hand.

She had mild intellectual disability (IQ 70) as assessed using the Korean Educational Developmental Institute-Wechsler Intelligence Scale for Children. Manual muscle testing revealed the hemiplegic left upper and lower limb muscles to have a strength grade of 4/5. Her right and left grip strengths (JAMAR Hydraulic Hand Dynamometer, Sammons Preston, PO Box 93,040 Chicago, IL 60673–3040 U.S.A.) were 25.0 kg (normal: 30.34 ± 5.24 kg) and 7.3, measured using a nine-hole pegboard (Sammons Preston, PO Box 93,040 Chicago, IL 60673–3040 USA), was defined as the time taken to place and remove nine headless pegs from holes in a 5-in. square pegboard. The patient took 18 s while using the right hand (normal: approximately 19 s) and 88 s while using the left hand (normal: approximately 21 s) at age 16 and 18 s for right hand and 62 s for left hands at age 21 [5]. The sensory assessment included five different modalities: light touch, sharp/dull, stereognosis, proprioception, and two-point discrimination. These modalities were scored as “intact”, “impaired” or “absent”. The sensation test revealed that stereognosis, proprioception, and two-point discrimination of the left hand were impaired. Mirror movement was assessed via three different tasks, as described by Woods and Teuber, namely, finger tapping, fist turning, and finger alternation. The mirror-movement scores for the right and left hands were 7 and 9, respectively (scores range from 0 to 12; higher scores indicate more mirror movement) [6].

### Conventional magnetic resonance imaging

**Fig. 1:**
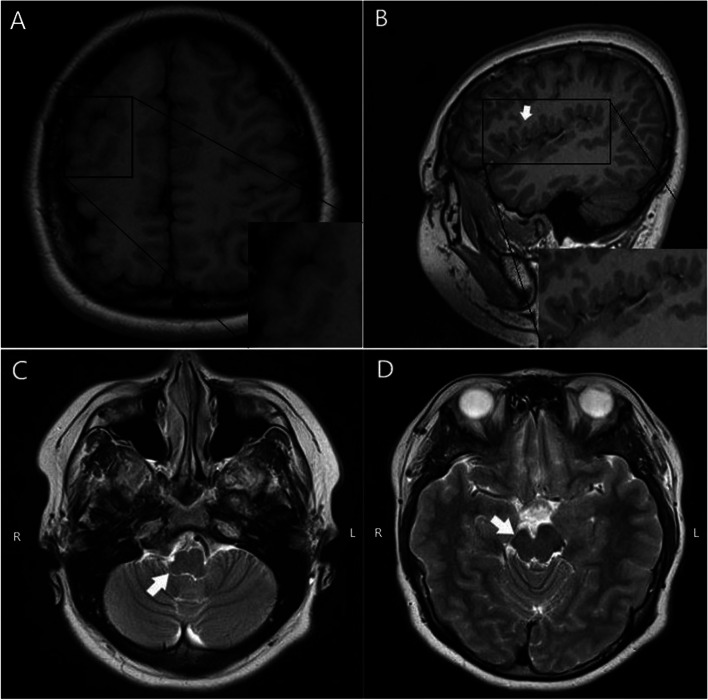
The axial T1-weighted image shows irregularities of the gray–white matter junction, and the cortex displays a nodular, bumpy appearance (**A**, arrow). MRI shows that the right half of the brain was smaller than the left half by visual comparison (**A**). The sagittal image reveals multiple small gyri in the sylvian fissure (**B**, arrow). The axial T2-weighted images show that the right half of the pons was significantly smaller than the left half (**C**, arrow), and the right cerebral peduncle of the midbrain was significantly smaller than the left (**D**, arrow)

Conventional magnetic resonance imaging (MRI) was acquired when our patient was 12 years old. Magnetic resonance (MR) images of the brain revealed that unilateral polymicrogyria affected the anterior cortical areas, including the perisylvian region, with relative sparing of the occipital cortex. The right halves of the brain and brainstem were significantly smaller than the left halves and exhibited irregularities of the gray–white matter junction, and the cortex showed a nodular and bumpy appearance (Fig. 1).

### Diffusion tensor imaging

DTI was performed when the patient was 16 years old. The equipment, technique, program and imaging parameters of DTI were the same as those described in our previous papers [7]. For fiber tracking of the CST, regions of interest (ROIs) were drawn on a color-coded two-dimensional FA map. A seed ROI was drawn in the CST portion of the anterior mid-pons. The DTI data of 16- and 15-year-old boys were used as controls (Control 1, 16 years old; Control 2, 15 years old). They came to the hospital with a simple headache, and DTI was taken, but no lesions on the brain were found.

**Fig. 2:**
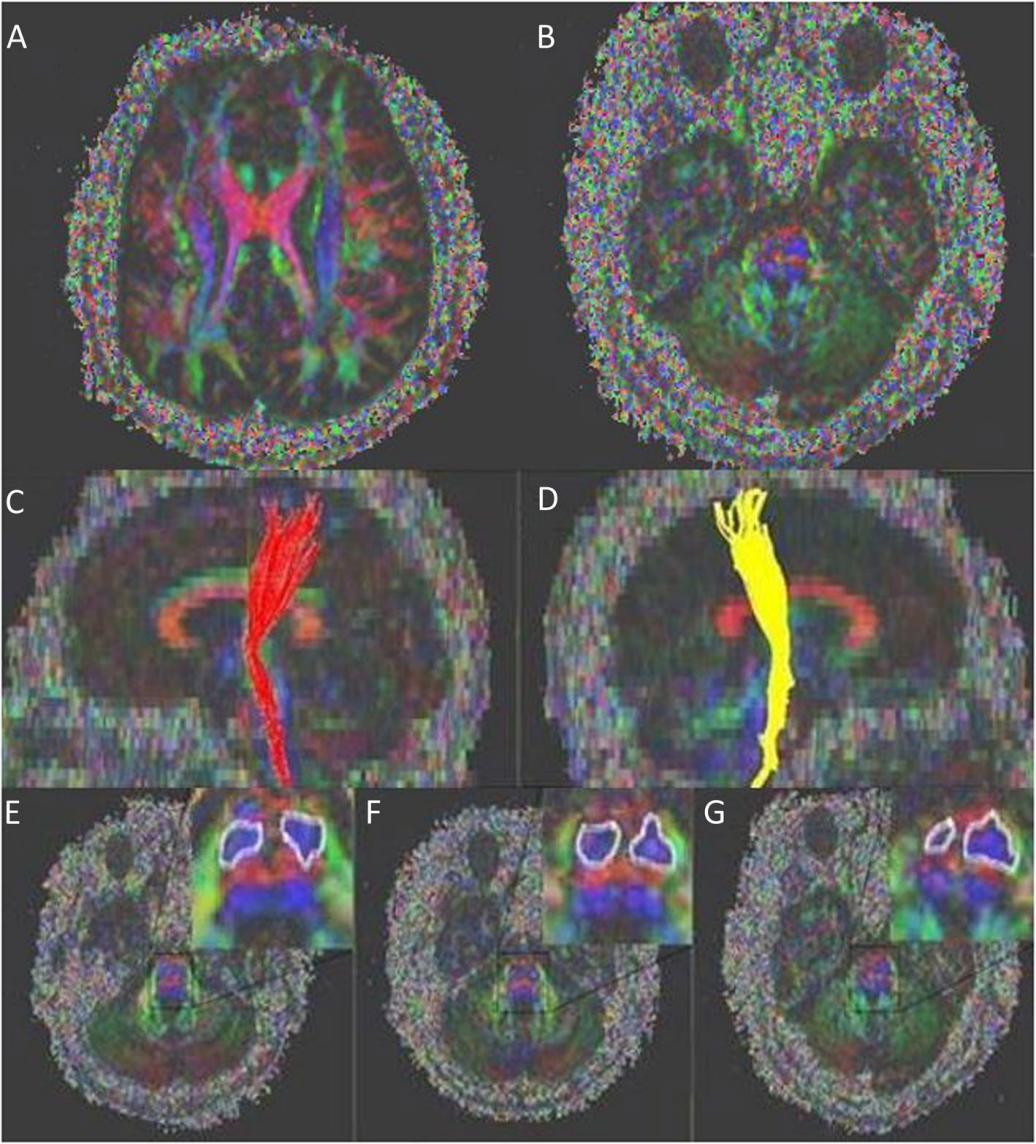
Axial color-coded fractional anisotropy map demonstrating the well-defined corticospinal tract (CST, blue) at the level of the cerebral cortex (**A**) and pons (**B**). The right corticospinal tract in the sagittal section (**C**). The left corticospinal tract in the sagittal section (**D**). The cross-sectional area of the corticospinal tract estimated with DTI. Control 1 (**E**), Control 2 (**F**), and our patient (**G**) featured symmetry indices of 0.86, 1.02, and 0.33, respectively

To assess the CST, the FA and apparent diffusion coefficient (ADC) of the bilateral pons were measured (Table 1). Although our patient was found to exhibit cortical dysplasia of right frontoparietal and sylvian fissure areas in conventional MRI, the connectivity of the CST was preserved up to the primary motor cortex (Fig. 2). We also used DTI to measure the cross-sectional area of the CST at the level of the pons in the controls and our patient. Figure 2 shows that our patient demonstrated significant asymmetry of the CST relative to the controls.

**Table 1:** Number of fibers, FA, and ADC values of the corticospinal tract

	Number of fibers		FA		ADC (× 10^−4^)	
	Right	Left	Right	Left	Right	Left
Control 1	680	710	0.64 ± 0.16	0.64 ± 0.12	6.90 ± 1.15	6.89 ± 1.23
Control 2	620	612	0.64 ± 0.16	0.63 ± 0.17	6.91 ± 1.42	6.90 ± 1.12
Patient	266	697	0.60 ± 0.17	0.66 ± 0.19	7.14 ± 1.36	6.90 ± 1.31

Values are presented as the mean ± standard deviation

*FA* Fractional anisotropy, *ADC* Average apparent diffusion coefficient

### Transcranial magnetic stimulation

TMS was performed using a Magstim 200 stimulator (Magstm, Whitland, Wales, UK) equipped with a 65-mm figure-eight coil and a Medtronic Keypoint® electromyography unit (Medtronic Inc., Skovlunde, Denmark) with a digitization rate of 10 kHz, a high-pass filter set to 100 Hz, and a low-pass filter set to 5 kHz. Motor evoked potentials (MEPs) of the first dorsal interosseous (FDI) muscles of both hands were recorded simultaneously. TMS mapping over the hand area of each cortex was obtained along a 2 × 2-cm grid at rest. The coil handle pointed backward and 45° away from the midline. Both hemispheres were assessed for ipsilateral or contralateral MEPs. For each detected MEP, the following parameters were determined: the optimal point (the scalp position at which reproducible muscle responses were elicited with the lowest stimulation intensity) and the motor threshold at rest (the minimum stimulation intensity that produced at least five MEPs exceeding 50 uV in 10 trials). The stimulation intensity was set to 110% of the motor threshold. Each site was stimulated four times and the shortest latency and the peak-to-peak amplitudes were used for analysis.

**Fig. 3:**
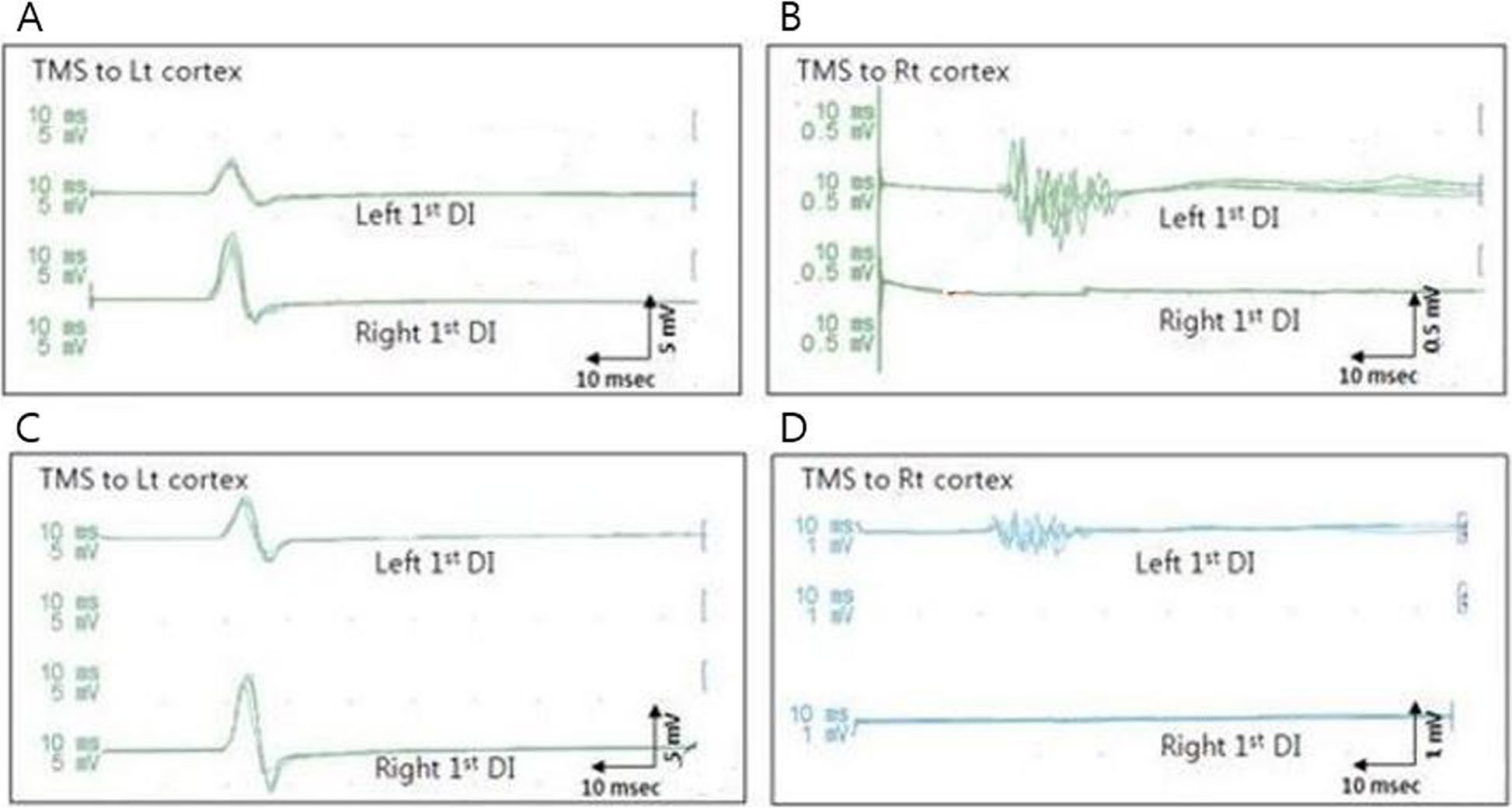
TMS at the ages of 16 (**A**, **B**) and 21 (**C**, **D**). The right and left 1st dorsal interosseous (DI) motor evoked potential responses to the transmagentic stimulation of the left primary motor cortex (**A**, **C**) and right primary motor cortex (**B**, **D**)

The TMS study was performed for our patient at ages16 and 2. The optimal stimulation site for the FDI muscles was identified at 3 cm lateral to the vertex on both hemispheres. The resting motor threshold at the optimal stimulation was 65 and 66% in the left and right hemispheres, respectively (normal motor threshold: 40%) [8] for contralateral MEPs and 58% in the left hemisphere for ipsilateral MEPs. Contralateral MEPs were evoked from both hands, but the ipsilateral MEPs were evoked only from the left hand (Fig. 3). When the left cortex was stimulated, we observed short-latency, long-duration ipsilateral MEPs on the patient’s paretic hand (latency, 18.6 ± 0.2 ms, age 16; 18.8 ± 0.0 ms, age 21) (amplitude, 2.57 ± 0.37 mV, age 16; 3.62 ± 0.42 mV, age 21) and long-latency, short-duration contralateral MEPs on the patient’s nonparetic hand (latency: 19.2 ± 0.2 ms, age 16; 20.0 ± 0.0 ms, age 21; amplitude: 5.76 ± 0.56 mV, age 16; 4.67 ± 0.90 mV, age 21) (normal latency: 20.6 ± 2.9 ms, amplitude 2.36 ± 1.23 mV) (Table 2) [9]. The paretic hand featured a long duration, polyphasic pattern of contralateral MEPs which persisted with age (Fig. 3).

**Table 2:** TMS at the ages of 16 and 21

Stimulation/Recording	Latency (ms)		Amplitude (uV)	
	Right FDI	Left FDI	Right FDI	Left FDI
A				
Left cortex	19.2 ± 0.2	18.6 ± 0.2	5.76 ± 0.56	2.57 ± 0.37
Right cortex	Not evoked	19.2 ± 0.2	Not evoked	0.57 ± 0.05
B				
Left cortex	20.0 ± 0.0	18.8 ± 0.0	4.67 ± 0.90	3.62 ± 0.42
Right cortex	Not evoked	20.0 ± 0.0	Not evoked	0.46 ± 0.09

TMS at the ages of 16 (A) and 21 (B)

Values are presented as the mean ± standard deviation

*TMS* Transcranial magnetic stimulation, *FDI* First dorsal interosseous

## Discussion and conclusions

To the best of our knowledge, our study is the first to use TMS and DTI to investigate changes in motor organization induced by unilateral polymicrogyria associated with ipsilateral brainstem atrophy. Previous studies of polymicrogyria have reported MRI and pathology findings corresponding to right unilateral cerebral hemisphere atrophy as well as TMS data associated with unilateral extensive cortical dysplasia with mirror movement [10]. However, neither study involved brainstem atrophy. Although MRI [2] and DTI [11] have been previously used to assess unilateral polymicrogyria, TMS not used in that study.

In this report, we document the case of a left hemiplegic patient with right hemispheric, unilateral polymicrogyria associated with ipsilateral brainstem atrophy. This patient’s paretic hand evinced ipsilateral corticospinal projections from the unaffected left hemisphere and contralateral corticospinal projections from the affected right hemisphere. These findings can be attributed to the concurrent bilateral projections to the paretic hand from the affected and unaffected hemispheres [12].

TMS revealed higher than normal resting motor thresholds in both cortices, which was thought to be due to topiramate (sodium channel blocker) usage by our patient. In previous studies, anticonvulsants depressed the excitability of motor pathways and elevated motor thresholds [13].

Prolonged duration of contralateral MEPs from the affected hemisphere is found in both the normative and pathological maturation of corticospinal tract projections [14]. The delayed latency of contralateral MEPs from the affected hemisphere is characteristic of the postischemic reorganization of central motor pathways [15, 16]. Contralateral MEPs in the paretic hand were polyphasic, indicating delayed electrophysiological maturation or the pathologic condition of the corticospinal motor pathways.

Based on several reports attributing polyphasic MEPs to the pathological alternations of the corticospinal pathways induced by demyelination and remyelination [17], we assumed that the contralateral polyphasic configuration of the MEP pattern of the affected hemisphere was caused by pathological alterations of the corticospinal pathways. Interestingly, the ipsilateral MEPs in the paretic hand showed patterns similar to those observed in the nonparetic hand, suggesting that ipsilateral MEPs from the paretic hand and contralateral MEPs from the nonparetic hand share the same neural substrate. Furthermore, we assumed that although ipsilateral MEPs are maintained, contralateral projections from the affected hemisphere are developed, probably due to brain plasticity [18]. If only one hemisphere is affected by a congenital lesion, the contralesional hemisphere features compensatory potential. The TMS data revealed reorganized ipsilateral projections from the contralesional hemisphere to the paretic hand.

We used DTI to visualize the CST, and the CST was objectively quantified through FA, ADC, and the number of fibers of DTI parameters. The DTI index reportedly correlates with the conventional MRI index in children with hemiplegia and provides a useful prognostic tool for anticipating upper-limb deficits [19]. Although the bilateral connectivity of the CST was preserved up to the primary motor cortex, we found fewer CST fibers on the affected side than on the unaffected side. Furthermore, the cross-sectional area of the CST at the level of the pons on the affected side was smaller than that on the unaffected side. These DTI findings reveal an inadequate CST volume. However, it is noteworthy that connectivity of the CST was preserved up to the primary motor cortex, possibly resulting in the decreased severity of our patient’s paretic hand motor functions.

Clinically, patients with hemiplegia can present with seizures and mild intellectual disability. Surprisingly, the motor functions of our patient were less impaired than expected based on the MRI findings, presence of hemibrainstem atrophy, and similar case reports. Despite extensive brain malformation and ipsilateral brainstem atrophy, our patient had less severe motor dysfunction and presented with involuntary mirror movements in her paretic hand, which has not been previously reported. Mirror movements in the paretic hand are considered to indicate ipsilateral corticospinal projections from the unaffected hemisphere and may suggest favorable motor outcomes in early brain injury [6].

Our patient can be classified as having pathologically acquired mirror movement when considering that mirror movement was visible from one hand at 5 years of age or older. There are two types of mirror movements, physiological and pathological. The former usually appears before the age of 5 years and is most commonly due to the fact that the corpus callosum is immature [20, 21]. After the age of 5, pathological mirror movements can be further divided into congenital and acquired mirror movements: the former features ipsilateral MEPs in both hands that can become dominant [22]; the latter is characterized by mirror movements in only one hand and the dominance of contralateral MEPs. However, when patients perform tasks with the paretic hand, mirror movements of the nonparetic hand are relatively weak, indicating that different motor tracts govern voluntary and mirror movements [6].

This study is subject to several limitations. First, we did not perform cytomegalovirus immunoglobulin M antibody titer or chromosomal studies. Additionally, a genetic study did not discriminate between polymicrogyria and other genetic disorders, and MRI was sufficient to diagnose polymicrogyria [23]. Second, DTI is particularly sensitive to artifacts generated by eddy currents, motion, and susceptibility that cause ghosting and geometric distortions [24]. While our system is equipped with powerful gradients and parallel imaging that yield good-quality DTI maps, some degree of shape distortion cannot be excluded. Third, the DTI parameters of two healthy controls of similar age to our patient were obtained. A greater number of healthy subjects of the same age are needed to reach more significant values of DTI parameters. Finally, as our data were derived from a case report, the prospective imaging of patients with unilateral polymicrogyria associated with ipsilateral brainstem atrophy is necessary to validate our findings.

## Data Availability

The datasets used and/or analyzed during the current study are available from the corresponding author on reasonable request.

## Abbreviations

TMS: Transcranial magnetic stimulation
DTI: Diffusion tensor imaging
CST: Corticospinal tract
FA: Fractional anisotropy
ADC: Apparent diffusion coefficient
ROI: Regions of interest
MEPs: Motor evoked potentials
FDI: First dorsal interosseous
MR: Magnetic resonance
